# Reliability and Validity of Slovenian Versions of IPAQ-SF, GPAQ, and EHIS-PAQ for Assessing Physical Activity and Sedentarism of Adults

**DOI:** 10.3390/ijerph19010430

**Published:** 2021-12-31

**Authors:** Kaja Meh, Vedrana Sember, Saša Đurić, Henri Vähä-Ypyä, Paulo Rocha, Gregor Jurak

**Affiliations:** 1 Faculty of Sports, University of Ljubljana, 1000 Ljubljana, Slovenia; vedrana.sember@fsp.uni-lj.si (V.S.); gregor.jurak@fsp.uni-lj.si (G.J.); 2 Liberal Arts Department, General Education, American University of the Middle East, Egaila 54200, Kuwait; sasa.duric@aum.edu.kw; 3 UKK-Institute, 33500 Tampere, Finland; henri.vaha-ypya@ukkinstituutti.fi; 4 Portuguese Institute of Sport and Youth, 1250-190 Lisbon, Portugal; paulo.rocha@ipdj.pt

**Keywords:** survey, questionnaire, self-report, accelerometer, measurement characteristics, sedentary behavior, lifestyle, public health

## Abstract

Health policies rely on physical activity (PA) and sedentary behavior data collected through PA questionnaires (PAQs). Validity of international PAQs varies among countries. Therefore, it is important to know the validity of the national versions of the PAQs to properly evaluate the results. We conducted a validation study of the Slovenian versions of the International PAQ Short Form (IPAQ-SF), the Global PAQ (GPAQ), and the PAQ used in the European Health Interview Survey (EHIS-PAQ) on 306 healthy adults. The most valid and reliable constructs in all tested were sedentary behavior and vigorous PA (VPA), however the criterion validity of these constructs was low (Spearman’s ρ 0.38–0.45 for sedentary behavior and 0.34–0.42 for VPA). Moderate to vigorous PA (MVPA) had low validity (0.26–0.29) despite being used as a standard measure of PA behavior. Participants over-reported MVPA for −9 to 64 min and underreported the sedentary behavior for more than two hours. The test-retest study found high reliability for sedentary behavior (0.69–0.81) and low to moderate reliability for PA behavior (0.42–0.76). The Slovenian versions of the observed PAQs are a useful tool for national PA surveillance, but for qualitative assessment of individual health-related PA behavior they should be combined with accelerometer-based devices.

## 1. Introduction

Regular physical activity and sedentariness are independent risk factors for several health conditions: cardiovascular disease and adiposity [1], all-cause mortality [2], type 2 diabetes [3], and some cancers [4]. They are also associated with metabolic [5], mental [6], and cognitive health [7]. Therefore, monitoring of physical activity and sedentary behavior is important both for individuals to assess their daily movement patterns and for society to evaluate the effectiveness of policies to combat non-communicable diseases. Consequently, the assessment of physical activity is rapidly evolving. As technology evolves, device-based measurement (e.g., accelerometers, wristbands, smart watches) is emerging. Such measurement is more valid and reliable compared to the use of physical activity questionnaires (PAQs) as a subjective method [8], but also more time-consuming and expensive [9]. As suggested by Pettee-Gabriel and colleagues (2012), methodological issues must be considered when selecting an appropriate method to assess physical activity [10]. Study, population, instrument, and activity characteristics influence the choice of measurement instruments. For this reason, PAQs are still widely used [11] and will remain so as they are the most suitable tool for epidemiological studies [12]. The measurement characteristics of different PAQs have already been tested in several nations and cultures [13] and their low to moderate validity and moderate to high reliability are well known [9,14].

PAQs have been a mainstay of measurement in most non-communicable diseases surveillance systems and epidemiological studies; however, their use is not unified. For example, a recent study of national surveillance systems for physical activity, sedentary behavior, and sport participation in the European Union [15] found that the International Physical Activity Questionnaire (IPAQ), with its long and short forms (IPAQ-LF and IPAQ-SF; [16]), prevails in the European Union (EU). Globally, the Global Physical Activity Questionnaire (GPAQ) is used in more than 120 countries worldwide and is used in surveys conducted by the World Health Organization (WHO) [17,18]. Still, wide variety of other PAQs is used in the EU. For example, the EU Eurobarometer uses the adapted IPAQ-SF [19], the European Health Interview Survey (EHIS) uses its own PAQ [20], Nordic countries use the Nordic Physical Activity Questionnaire [21], and many member states have their own national questionnaires [15].

Interestingly, previous findings have shown that the validity of the most commonly used international PAQs varies between different countries or regions. For example, in a study of 12 countries, Craig and colleagues (2003) found differences in the reliability and validity of IPAQ-SF and IPAQ-LF [22]. Concurrent validity showed low to high correlations ranging from Spearman’s ρ = 0.43 (Portugal) to 0.88 (The Netherlands) for physical activity and from Spearman’s ρ = 0.69 (United States) to 0.96 (Finland) for sitting. Additionally, differences in the test-retest reliability of IPAQ-SF were found between urban participants from the United States (Spearman’s ρ = 0.89) and South Africa (Spearman’s ρ = 0.69) and between rural participants from South Africa (Spearman’s ρ = 0.32) [22]. More importantly, differences in the criterion validity of IPAQ-SF were found between European countries. For moderate to vigorous physical activity (MVPA), values vary from Spearman’s ρ = 0.13 in United Kingdom [23] to Pearson’s ρ = 0.47 in Spain [24]. Similar variability was observed for concurrent validity of vigorous physical activity (VPA) measured with GPAQ. In European countries, values ranged from Spearman’s ρ = 0.51 in Austria [25] to Spearman’s ρ = 0.86 in France [26]. Results for criterion validity of VPA measured with the GPAQ were not scattered, yet they were low to moderate; Spearman’s ρ = 0.31 in Germany [27] to Spearman’s ρ = 0.64 in a multinational study in Belgium, Spain, and the United Kingdom [28]. Interestingly, only one reliability and validity study of EHIS-PAQ has been published to date [20], although all member states regularly use national versions of this questionnaire as part of the EHIS [29]. Detailed overview of reliability and validity results from European countries can be found elsewhere [14,30].

As European and national health policies and programs are based on the physical activity and sedentary behavior data collected using the described PAQs, the use of validated measures is necessary. Many of the EU countries have not tested the reliability and validity of their national versions of the widely used international PAQs [14,30] and Slovenia is among them. Therefore, within the European Physical Activity and Sports Monitoring System (EUPASMOS) project, we prepared a study on the reliability and validity of Slovenian versions of EHIS-PAQ, IPAQ-SF, and GPAQ, aiming to fill this gap in Slovenia.

## 2. Materials and Methods

### 2.1. Study Design and Participants

We recruited participants for the study using a snowballing method. Through 7th and 9th graders from nine Slovenian primary schools from rural and urban areas in central Slovenia, we contacted their parents, grandparents, and adult siblings. Only healthy participants whose health was not affected by physical activity were included. Participants self-assessed their health status. If participants reported any health problems that could affect their daily physical activity (e.g., injuries or a chronic health problems), a kinesiology professional assessed their health status and decided if they could participate in the study. Permission for conducting the study was obtained from the Faculty of Sport in Ljubljana in accordance with the Declaration of Helsinki (No: 6:2020-274). The data of the present study were obtained as part of the European project EUPASMOS No. 590662-EPP-1-2017-1-PT-SPO-SCP.

A total of 399 adults were enrolled in the initial sample (40.6% male, mean age = 41.16, SD = 14.35, mean BMI = 25.23, SD = 4.37), but due to incomplete data 93 participants were excluded from the analysis (invalid questionnaire data or invalid accelerometer data). We detected no bias between included and excluded participants; there were no differences in age, BMI, and accelerometer measured MVPA. Finally, 306 healthy participants older than 18 years were included in our study (39.2% male, mean age = 40.57, SD = 14.35, mean BMI = 24.84, SD = 4.39). Among them, 181 participants were part of the test-retest study (39.2% male, mean age = 36.30, SD = 13.88, mean BMI = 24.33, SD = 4.08). A post hoc power analysis was then performed and revealed that with our sample size correlation coefficient of 0.3 could be detected at *p* < 0.01, one tailed at a power of 0.99.

During the first visit, reliability study participants completed the Slovenian versions of all three PAQs in randomized order. We provided a quiet and comfortable environment in a computer room, where participants had ample time to complete the online versions of all three PAQs. It took participants approximately 15 min to complete all PAQs. Accelerometers were then given to all participants, and they were familiarized with their use. Participants were instructed to wear the accelerometers all the time for the next seven consecutive days, except while doing different water activities (e.g., swimming, showering, going to the sauna). After 7 days, all participants came back for the second visit. They returned the accelerometers and continued with the anthropometric measurements (height, weight). At the end, all participants completed all three PAQs in randomized order.

### 2.2. Subjective Measures of Physical Activity

Three international PAQs most commonly used in the EU were used to assess physical activity: IPAQ-SF, GPAQ, and EHIS-PAQ [14].

IPAQ-SF contains 7 items assessing physical activity and sitting time in the last 7 days. Participants are assessing frequency and duration of their walking, MPA, VPA and duration of sitting on a regular day. In the 12-country reliability and validity study of IPAQ, they found a moderate to high reliability of the questionnaire (Spearman’s ρ = 0.66–0.87 for total physical activity and Spearman’s ρ = 0.50–0.95 for sitting) [22]. Criterion validity was tested against CSA motion detector MTI and was very low to low for physical activity (Spearman’s ρ = 0.02–0.47) and sitting (Spearman’s ρ = 0.12–0.46).

The GPAQ contains 16 items assessing physical activity in a typical week in three domains: work, transport, and recreation. Participants assess the intensity of physical activity, frequency, and duration. One item is used to assess the amount of time spent sitting on a typical day. Nine country reliability and validity study showed moderate to high reliability results (Spearman’s ρ = 0.67–0.73 for physical activity and Spearman’s ρ = 0.68 to 0.73 for sedentary) [17]. Criterion validity measured against CSA motion detector MTI was very low to low (Spearman’s ρ = −0.03–0.23 for MPA, Spearman’s ρ = 0.23–0.26 for VPA and Spearman’s ρ = −0.02–0.4 for sedentary).

EHIS-PAQ questions are also based on a typical week with 10 items. Participants assess three domains of physical activity (work, transport, and health enhancing), two dimensions of physical activity (frequency and duration), and duration of time spent sitting on a typical day. With EHIS-PAQ, total MVPA is not calculated; instead, moderate to vigorous aerobic recreational activity item is used as a measure of MVPA recommended by WHO. One reliability and validity study of EHIS-PAQ was published to date and showed moderate to high test-retest reliability for physical activity items (ICC = 0.51–0.73) and very low to low criterion validity against ActiGraph GT3X (ICC = 0.06–0.44) [20].

We used the forward-backward method to translate the PAQs recommended by WHO [31]. Two independent translators interpreted the PAQs from English into Slovenian and two other translators back into English. We compared the two English versions and decided on the best translation. The translations were very similar, and we decided on the best version for each of the questionnaires.

### 2.3. Accelerometer Measured Physical Activity

A tri-axial accelerometer (RM42, UKK Terveyspalvelut Oy, Tampere, Finland) was worn on the right hip during waking hours and on the non-dominant wrist during bedtime. Acceleration data were collected within a range of ±16 G at a sampling rate of 100 Hz and stored on a hard disc for further analysis. The analysis of physical activity was based on the mean amplitude deviation (MAD) in six-second epochs [32]. MAD has been found a valid indicator of incident oxygen consumption during locomotion [33]. For each epoch, the MAD values were converted to METs (3.5 mL/kg/min of oxygen consumption). The epoch-wise MET values were further smoothed by calculating an exponential moving average for each epoch time point [34]. The smoothed data were analyzed in 6-s epochs and the physical activity cut points were set as follows: 3.0 METs ≤ MPA < 6.0 METs and VPA ≥ 6.0 METs.

Sedentary behavior (sitting and lying) and standing were identified for the epochs in which the predicted MET value was less than 1.5. The orientation of the accelerometer with respect to the gravity vector was taken as reference, and the angle for posture (APE) estimation was determined from the orientation of the accelerometer with respect to the reference vector [35]. Posture was classified as standing if the angle for posture was less than 11.6°, sitting if the angle for posture was between 11.6° and 72.0°, and lying if the angle for posture was greater than 72.0°. The six-second epoch-wise values representing posture were also smoothed by a one-minute exponential moving average.

A valid day was defined as having at least 600 min of monitor wear. Participants had to wear accelerometer at least 4 valid wear days, one of which had to be weekend day, to be included in the study.

### 2.4. Anthropometry

Height (to the nearest 0.1 cm) and weight (to the nearest 0.1 kg) were measured while participants were without shoes and in light clothing using the Seca 799 electronic scale (Seca Deutschland, Hamburg, Germany). We calculated body mass index (BMI) from height and weight and categorized participants into four categories based on WHO [36]: underweight (BMI < 18.5), healthy weight (18.5 < BMI < 24.9), overweight (25 < BMI < 29.9), and obese (BMI > 30).

### 2.5. Statistical Analysis

Statistical analysis was performed using IBM SPSS 27 software (Armonk, NY: IBM Corp) and Microsoft Excel. The normality of the data was tested using the Kolmogorov-Smirnov test. Since the data were not normally distributed, we used Spearman’s rank correlations to test the reliability and validity of the questionnaires. To interpret reliability and validity, we used the following criteria: ≤0.29 very low, 0.30–0.49 low, 0.50–0.69 moderate, 0.70–0.89 high, and greater than 0.90 very high validity [37]. We created a Bland-Altman plot to determine the agreement between the accelerometer and each of the PAQs.

## 3. Results

There were some differences in BMI: more overweight and obese individuals were among males (56.3%) than among females (33.7%) (Table 1), most of whom were in the normal BMI category (64.3%). There were also statistically significant gender differences in MPA, VPA, and MVPA measured with accelerometer UKK RM42 and in self-reported VPA (IPAQ-SF and GPAQ).

The metadata on the PAQs showed that participants spent from two to almost four minutes to complete each of the PAQs. On average it took participants 2 min and 24 s for IPAQ-SF, 3 min and 21 s for the GPAQ, and 2 min and 58 s for the EHIS-PAQ.

Reliability was tested using the test-retest method (Table 2). All results were statistically significant (*p* ≤ 0.001) and showed “low to high” correlations between measurements. With the exception of IPAQ-SF MPA, GPAQ work VPA, and leisure MPA, all correlations were higher than 0.5 and were therefore considered “moderate” [37]. IPAQ-SF and GPAQ had the highest correlations for sedentary behavior (Spearman’s ρ = 0.808 and 0.814, respectively). For EHIS-PAQ, we found the highest test-retest correlations for cycling (Spearman’s ρ = 0.809). For each PAQ, we assessed internal consistency with the Cronbach’s alpha coefficient twice, with and without the sedentary behavior question. The IPAQ showed a “rather reliable” correlation with the sedentary behavior question (Cronbach’s α = 0.297) and a “reliable” without it (Cronbach’s α = 0.685). Similar results were found for the GPAQ: “rather reliable” with the sedentary behavior question (Cronbach’s α = 0.235) and “reliable” without it (Cronbach’s α = 0.669). We found no large differences in EHIS-PAQ, when we excluded the sedentary behavior question. Internal consistency was “rather reliable” with the sedentary behavior question (Cronbach’s α = 0.304) and stayed “rather reliable” without it (Cronbach’s α = 0.310).

The results of the concurrent validity are presented in Table 3. Most of the correlations between the PAQs were very low to low, with some exceptions. For example, sedentary behavior was highly and statistically significantly correlated (Spearman’s ρ = 0.777, 0.772, and 0.857). VPA was moderately correlated (Spearman’s ρ = 0.634), as was MVPA (Spearman’s ρ = 0.597) between IPAQ-SF and GPAQ. There were more relevant and “low to moderate” correlations between GPAQ and IPAQ-SF (e.g., MPA, VPA). In contrast, EHIS-PAQ had no “moderate” correlations with either of the other two PAQs for physical activity, except for the walking item, which correlated with IPAQ-SF walk. “Close to moderate” correlations were found for transport related physical activity (GPAQ transport/EHIS-PAQ walk Spearman’s ρ = 0.497, IPAQ-SF walk/EHIS-PAQ walk = 0.558). 

The criterion validity correlations between the PAQs and the UKK RM42 accelerometer are presented in Table 4. We found “low to moderate” and significant correlations for VPA and sedentary behavior. For GPAQ, leisure-time VPA correlated Spearman’s ρ = 0.534 with UKK RM42 while VPA = 0.415. IPAQ-SF VPA had Spearman’s ρ = 0.342 with VPA measured by UKK RM42, while EHIS-PAQ showed no statistically significant correlations for moderato to vigorous recreational activity. For sedentary behavior, IPAQ-SF Spearman’s ρ was 0.454 while for GPAQ was 0.4 and EHIS-PAQ was 0.376.

**Figure 1:**
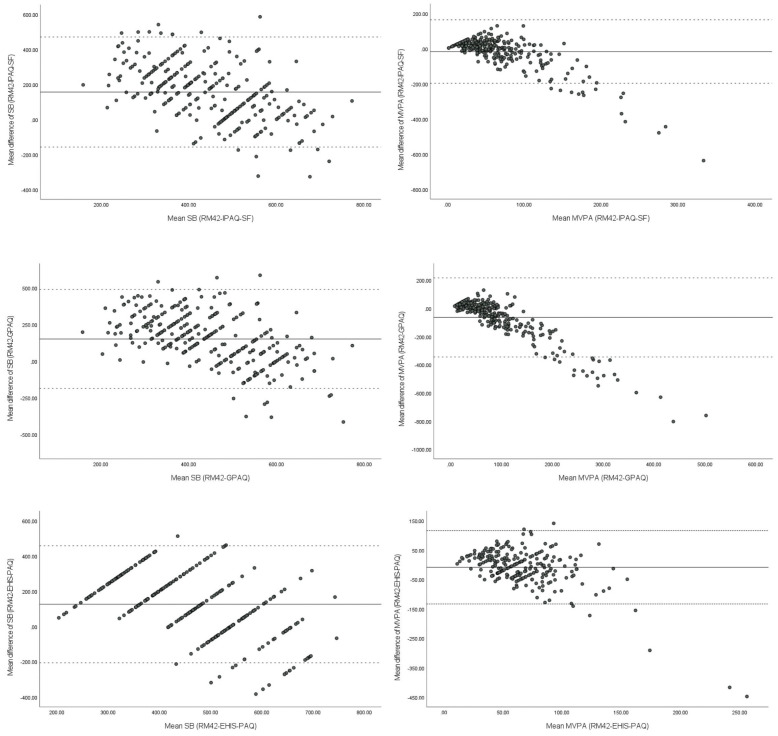
Bland-Altman plots for the PAQs and UKK RM42 accelerometer according to sedentary behavior and MVPA (min/day) with 95% limit of agreement.

With Bland-Altman plot (Figure 1), we present the differences between data on MVPA and sedentary behavior collected with the UKK RM42 accelerometer and PAQs. One-sample T-tests revealed significant differences between the accelerometers and all three questionnaires for sedentary behavior and MVPA for IPAQ-SF and GPAQ (GPAQ: *p* < 0.000, IPAQ-SF MVPA: *p* < 0.002). Participants underestimated their sedentary behavior on all three PAQs. For sedentary behavior, average difference was lowest for EHIS-PAQ at 125 ± 169 min, while participants underestimated their sitting time by about 2.5 h with IPAQ-SF and GPAQ (IPAQ-SF = 157 ± 160 min; GPAQ = 151 ± 172 min). On the other hand, participants overestimated their MVPA with IPAQ-SF and GPAQ; the average difference for the IPAQ-SF was 17 ± 92 min, followed by GPAQ with 64 ± 143 min). With EHIS-PAQ, participants on average underestimated their moderate to vigorous recreational activity , but the difference was close to zero (−9 ± 64 min on average).

According to the WHO guidelines on physical activity and sedentary behavior, 92.5% of participants achieved the recommended amount of MVPA. The figures were lower for the PAQs; 89.5% of participants were considered sufficiently active for IPAQ-SF, 87.3% for GPAQ and 48% for EHIS-PAQ.

## 4. Discussion

This study examined the reliability and validity of the Slovenian versions of EHIS-PAQ, IPAQ-SF, and GPAQ. The main finding of the study is that the most valid and reliable constructs in all tested PAQs were sedentary behavior and VPA, but the criterion validity of these constructs was low (Spearman’s ρ = 0.38–0.45 for sedentary behavior and 0.34–0.42 for VPA). The second important finding is that participants over-reported MVPA for 17 to 64 min and underreported the sedentary behavior for more than two hours with selected PAQs. Third, the GPAQ generally showed the highest criterion validity among observed PAQs, especially for VPA (Spearman’s ρ = 0.415).

The test-retest reliability results of all three PAQs were adequate (low to high correlations); the alpha coefficient also showed good internal consistency of IPAQ-SF and GPAQ and moderate internal consistency of EHIS-PAQ. Concurrent validity was high and significant for the sedentary behavior items between all three PAQs; comparable results were obtained in a recent meta-analysis for IPAQ-SF and GPAQ [30]. Compared to the UKK RM42 accelerometer physical activity data, PAQs showed lower performance. As expected, one of the most valid constructs in all selected PAQs was sedentary behavior. However, the associations in our study were low (0.38–0.45), while other studies found moderate to high correlations [30]. Nevertheless, the correlation coefficients for sedentary behavior in our study were still higher than in the two previous research that used the same device. In the Hungarian study, the Spearman’s ρ for IPAQ-SF sedentary time was 0.255 compared to the UKK RM42 accelerometer (*p* < 0.001) [38] and in the French study, the for GPAQ Spearman’s ρ was 0.26 (*p* < 0.005) [39]. Notably, results of all constructs in our study were higher compared to the two previous studies from France and Hungary: sedentary—Slovenia 531, Hungary 510, and France 481 min/week; MPA—Slovenia = 405, Hungary 366, and France 359 min/week; VPA—Slovenia 42, Hungary 22, and France 5 min/week.

The most valid constructs in all tested PAQs were sedentary behavior and VPA, which is consistent with other findings [22,26,27]. Related to this, we found higher correlations between VPA items compared to MPA items, which was also found in previous studies [23,26]. A possible explanation for these results may be sought in the constructs. The MPA and VPA constructs are based on descriptions of different physical activity intensities, and it is possible that the VPA descriptions are more accurate and easier to understand. Descriptions of bodily sensations during physical activity are highly subjective and every individual has their own scale of what is an increased heartbeat and when breathing becomes heavy. It is a challenge to create more objective descriptions of the body’s physiological responses. We believe that the descriptions used in the PAQs are appropriate, yet the subjectivity of these descriptions should be considered when using the PAQs and interpreting the data. We assume that individuals with better physical fitness will be better able to assess their MVPA, since their physiological response to physical activity is similar to physical activity intensity categorization, based on MET values.

Criterion validity for physical activity was generally highest for the GPAQ, a little better than IPAQ-SF. In addition, the GPAQ also showed higher criterion validity results for the domain-specific physical activity (work, transport, and leisure). It is known that longer and more detailed PAQs tend to be more valid [22], as detailed and domain-specific questions help participants recall physical activity. It is easier to recall physical activity in segments rather than remembering the entire MPA or VPA. The GPAQ contains more questions (16 questions) compared to IPAQ-SF (7 questions) and these questions are also domain specific, which can help participants recalling their daily or weekly physical activity for each domain. With nine additional questions, it took participants less than one minute more to complete the online version of the GPAQ compared to IPAQ-SF. We found particularly high criterion validity for the GPAQ leisure VPA (Spearman’s ρ = 0.534, *p* < 0.001), which represented the highest results from Slovenian data. Accelerometer-measured VPA was also higher compared to other studies using the UKK RM42 accelerometer [38,39]. Slovenians are among the most physically active and sporty nations in the EU [40], which could be the reason for the higher VPA compared to Hungarians and French. The results suggest that, given the increasing sedentariness combined with changes in the work environment, more and more people are being active in their leisure time [41,42]. As our results show, work related physical activity was not statistically significantly correlated with accelerometer-measured physical activity, but leisure VPA and transport VPA were. As lifestyles and workplaces are changing, so do the physical demands of workplaces. Physically demanding jobs may include lifting heavy objects, which was not correctly measured by the accelerometer. In addition, participants may subjectively assess their work as physically demanding (perhaps compared to other, more sedentary jobs), but their physical activity may not be intense enough to fall within the MVPA range. Previous research has also shown that highly sedentary people can be very physically active [5,43], and for these, domain-specific questions can help to recall physical activity [44]. In EU countries, work related physical activity is no longer as common anymore since people are mostly doing sedentary work. Therefore, more domain-specific questions can also highlight the changes in physical activity and sedentary behavior and provide more contextual information.

Despite slightly higher correlations compared to previous studies using the same instruments, our results showed that participants overestimated their MVPA with IPAQ-SF and GPAQ, but not with EHIS-PAQ (moderate to vigorous recreational activity reported). It has been previously proven that participants tend to over-report physical activity when using PAQs [45]. The systematic review of this problem found an average MVPA overestimation of 106%, when using IPAQ-SF [45], while our result found an overestimation of 29% when using IPAQ-SF, with GPAQ overestimation was higher, but on the other hand participants underestimated their PA when using EHIS-PAQ. WHO recommends 150 min of MVPA per week for adults to achieve significant health benefits [46]. According to our study, 92.5% of participants achieved this amount of MVPA based on the UKK RM42, while 89.5% of participants were sufficiently active according to the IPAQ-SF self-assessment, 87.3% according to the GPAQ, and 48% according to EHIS-PAQ. These overall low discrepancies indicate that PAQs are an appropriate tool for national physical activity surveillance systems to determine the level of physical activity in the population. However, problems with validity and over=reporting should be considered when examining individual physical activity behavior. Several previous studies have already pointed out the problem of over-reporting [47,48] and validity [14,30]; therefore, the results of PAQs should not be used as a measure of individuals’ health-related physical activity without additional information from objective measures.

Our results show greater similarity between IPAQ-SF and the GPAQ, as the concurrent validity between the two is the highest and both have similar criterion validity. Although EHIS-PAQ was developed specifically for the EU and its member states to outdo the disadvantages of PAQs [49], its results in our study on the Slovenian population were not better (MVPA/sport participation = 0.063) and lower compared to the results of the criterion validity study results from Germany, which used ActiGraph GT3X (MVPA = 0.32). Nevertheless, we believe that the question about “moderate to vigorous aerobic recreational activity” and the specific question about muscle-strengthening activities are important for physical activity surveillance. Muscle-strengthening activities are included in the WHO physical activity recommendations [46] and are not included in the most popular and widely used PAQs. The “moderate to vigorous aerobic recreational activity” item highly correlated with leisure physical activity from the GPAQ, once again demonstrating the need for domain-specific questions in PAQs.

The results of our study show that problems with over-reporting of physical activity and under-reporting of sedentary behavior also exist in the Slovenian sample of adults. We conclude that domain-specific physical activity questions can help with recall of physical activity and improve the validity of PAQs. Therefore, we would recommend the use of the GPAQ in Slovenian population. However, measuring physical activity using accelerometers remains more valid and reliable. Additional questions about muscle-strengthening activities not captured by accelerometers could outperform the accelerometer and provide additional information about individuals’ physical activity.

## 5. Strengths and Limitations

This is the first study to compare the reliability and validity of three different national versions of popular international PAQs using an objective measure. Comparing different PAQs provides data on their measurement characteristics and is a great way to compare differences between specific characteristics. In addition, this is the second study to validate the national version of EHIS-PAQ, which is, however, used in the EU as the primary data source for physical activity. With this study, we also validated for the first time IPAQ-SF, GPAQ, and EHIS-PAQ on a Slovenian sample. The main limitation arises from the sample. Since the PAQs are based on the subjective assessment of individual effort when performing physical activity, they could be influenced by the physical fitness of the participants. Therefore, variability in participants’ physical fitness may influence differences between subjective and objective measures of physical activity. Secondly, age distribution of participants was not representative. Most of the participants were younger than 50 years old, and therefore results of this study cannot be applied to all adults in general. According to previous findings and due to the lack of older participants in our sample, the reliability and validity of the presented PAQs might be lower in participants over 50 years of age. It could also be that physical activity is over-reported, so results for older participants should be interpreted with caution. This limitation is also found in previous validation studies. Therefore, more emphasis should be placed on appropriate sampling to avoid error. In addition, specific characteristics of the sample could affect the validity results, considering that fitter participants could understand the descriptions of physical activity intensity more accurately. The third limitation is associated with the MAD algorithm for the analysis of accelerometer data, which is validated for bipedal activities. Therefore, the intensity of activities of other types, such as cycling, is likely to be underestimated. As a result, the volume of VPA might also be underestimated. However, MAD algorithm has demonstrated a high comparability for the ActiGraph counts [50] and has shown a very strong association with VO_2_ during ambulatory activities [33]; moreover, similar problems with the measurement of VPA have been highlighted in other studies that used other algorithms for accelerometer data [51,52].

## 6. Conclusions

The comparison of the three most frequently used PAQs in the EU has shown that their reliability and validity in the Slovenian sample is similar to previous studies, yet validity results are still low to moderate. The present work highlights the differences between the subjective methods used to assess physical activity, in the same sample. We found that more detailed and domain-specific questionnaires provide a more valid measurement, and thus the measurement of VPA is more valid. Our findings confirm that PAQs can be a valid tool for non-communicable diseases surveillance systems in epidemiological studies to determine the percentage of individuals who are sufficiently active according to the WHO guidelines.

## Figures and Tables

**Table 1:** Descriptive statistics of the sample.

		Male		Female		Total	
Age Groups (N)	18–34	38	31.7%	60	32.3%	98	32.0%
	35–49	62	51.7%	87	46.8%	149	48.7%
	50–64	14	11.7%	21	11.3%	35	11.4%
	65–84	6	5.0%	18	9.7%	24	7.8%
BMI Category (N)	<18.5	0	0.0%	3	1.9%	3	1.2%
	18.5–24.9	45	43.7%	101	64.3%	146	56.2%
	25–29.9	42	40.8%	39	24.8%	81	31.2%
	>30	16	15.5%	14	8.9%	30	11.5%
MPA (min/week)	UKK RM42	451.7 (191.4)		364.0 (180.9)		405.3 (190.5)	
	IPAQ-SF	277.2 (397.6)		261.3 (471.4)		266.8 (442.6)	
	GPAQ	542.9 (780.2)		602.0 (828.8)		577.8 (807.9)	
VPA (min/week)	UKK RM42	50.5 (65.7)		34.9 (47.0)		42.2 (56.9)	
	IPAQ-SF	310.2 (389.7)		191.8 (242.4)		237.3 (313.2)	
	GPAQ	407.3 (555.9)		189.9 (282.8)		277.2 (425.6)	
MVPA (min/week)	UKK RM42	455.5 (209.1)		351.2 (178.6)		392.1 (197.5)	
	IPAQ-SF	587.4 (693.3)		453.1 (586.6)		504.9 (632.5)	
	GPAQ	950.1 (181.1)		791.9 (896.3)		854.4 (1019.3)	
	EHIS-PAQ	263.1 (225.8)		230.1 (204.9)		243.5 (213.8)	
Sitting (min/day)	UKK RM42	531.2 (105.5)		513.8 (103.0)		520.6 (104.2)	
	IPAQ-SF	362.0 (188.9)		372.2 (165.6)		366.4 (175.7)	
	GPAQ	369.0 (193.9)		385.9 (172.5)		370.1 (182.4)	
	EHIS-PAQ	405.6 (183.2)		395.6 (172.1)		395.8 (175.3)	

**Table 2:** Test-retest reliability of IPAQ-SF, GPAQ, and EHIS-PAQ.

			Retest																	
			IPAQ-SF						GPAQ								EHIS-PAQ			
			VPA	MPA	Walk	SB			Work VPA	Work MPA	Transport	Leisure VPA	Leisure MPA	SB			Walk	Cycle	MV Aerobic Recreational Activity	SB
Test	IPAQ-SF	VPA	0.648 *				GPAQ	Work VPA	0.466 *						EHIS-PAQ	Walk	0.671 *			
		MPA		0.461 *				Work MPA		0.626 *						Cycle		0.809 *		
		Walk			0.566 *			Transport			0.673 *					SP			0.472 *	
		SB				0.808 *		Leisure VPA				0.764 *				SB				0.694 *
								Leisure MPA					0.424 *							
								SB						0.814 *						

* *p* ≤ 0.001. All results are presented as minutes/day. Notes: IPAQ-SF = International Physical Activity Questionnaire—Short Form; GPAQ = Global Physical Activity Questionnaire; EHIS-PAQ = European Health Interview Survey—Physical Activity Questionnaire; VPA = vigorous physical activity; MPA = moderate physical activity; SP = sport participation; SB = sedentary behavior; MV = moderate to vigorous.

**Table 3:** Concurrent validity of IPAQ-SF, GPAQ, and EHIS-PAQ.

		GPAQ									IPAQ-SF				
		Work VPA	Work MPA	Transport	Leisure VPA	Leisure MPA	MPA	VPA	MVPA	SB	VPA	MPA	Walk	MVPA	SB
EHIS-PAQ	Walk	0.089	0.142 *	0.497 **	0.077	0.264 **	0.219 **	0.133 *	0.227 **	−0.239 **	0.114 *	0.118 *	0.558 **	0.140 *	−0.194 **
	Cycle	0.029	0.063	0.422 **	0.118 *	0.147 *	0.118 *	0.105	0.117 *	−0.145 *	0.092	0.216 **	0.120 *	0.187 **	−0.154 **
	MV aerobic recreational activity	0.122	0.062	0.021	0.175*	0.001	0.034	0.180*	0.158*	−0.001	0.287 **	0.0095	0.025	0.249 **	−0.013
	SB	−0.158 **	−0.321 **	−0.145 *	0.061	−0.208 **	−0.343 **	−0.035	−0.307 **	0.777 **	−0.088	−0.170**	−0.178 **	−0.189 **	0.772 **
IPAQ-SF	VPA	0.324 **	0.252 **	0.158 **	0.541 **	0.183 **	0.277 **	0.634 **	0.506 **	−0.159 **					
	MPA	0.244 **	0.361 **	0.183 **	0.164 **	0.373 **	0.450 **	0.292 **	0.483 **	−0.235 **					
	Walk	0.137 *	0.262 **	0.382 **	0.024	0.322 **	0.380 **	0.130 *	0.363 **	−0.215 **					
	MVPA	0.341 **	0.392 **	0.187 **	0.376 **	0.318 **	0.446 **	0.523 **	0.597 **	−0.241 **					
	SB	−0.207 **	−0.374 **	−0.169 **	0.042	−0.151 **	−0.356 **	−0.089	−0.329 **	0.857 **					

* *p* ≤ 0.05; ** *p* ≤ 0.01. All results are presented as minutes/day. Notes: IPAQ-SF = International Physical Activity Questionnaire—Short Form; GPAQ = Global Physical Activity Questionnaire; EHIS-PAQ = European Health Interview Survey—Physical Activity Questionnaire; VPA = vigorous physical activity; MPA = moderate physical activity; SB = sedentary behavior; MV = moderate to vigorous.

**Table 4:** Criterion validity of IPAQ-SF, GPAQ, and EHIS-PAQ against accelerometer UKK RM42.

	IPAQ-SF						GPAQ									EHIS-PAQ			
		VPA	MPA	Walk	MVPA	SB	Work VPA	Work MPA	Transport	Leisure VPA	Leisure MPA	MPA	VPA	MVPA	SB	Walk	Cycle	MV Aerobic Recreational Activity	SB
UKK RM42	VPA	0.342 **	0.176 **	−0.043	0.262 **	0.090	0.065	−0.063	0.109	0.534 **	0.119 *	0.010	0.415 **	0.165 **	0.115 *	0.042	0.102	0.067	0.114 *
	MPA	0.258 **	0.179 **	0.313 **	0.243 **	−0.143 *	0.050	0.118 *	0.293 **	0.291 **	0.177 **	0.181 **	0.249 **	0.234 **	−0.115 *	0.261 **	0.125 *	0.071	−0.153 **
	MVPA	0.319 **	0.209 **	0.304 **	0.289 **	−0.132 *	0.060	0.094	0.305 **	0.380 **	0.195 **	0.180 **	0.321 **	0.263 **	−0.098	0.257 **	0.142 *	0.063	−0.129*
	SB	−0.049	−0.202 **	−0.233 **	−0.169 **	0.454 **	−0.083	−0.324 **	−0.115 *	0.070	−0.225 **	−0.350 **	0.009	−0.280 **	0.400 **	−0.151 **	−0.093	0.08	0.376 **

* *p* ≤ 0.05; ** *p* ≤ 0.01. All results are presented as minutes/day. Notes: RM42 = RM42 triaxial accelerometer; IPAQ-SF = International Physical Activity Questionnaire—Short Form; GPAQ = Global Physical Activity Questionnaire; EHIS-PAQ = European Health Interview Survey—Physical Activity Questionnaire; VPA = vigorous physical activity; MPA = moderate physical activity; MVPA = moderate to vigorous physical activity; SB = sedentary behavior; MV = moderate to vigorous.

## Data Availability

The data presented in this study are available in SPSS file in Supplementary Materials.

## Supplementary Materials

The following supporting information can be downloaded at: https://www.mdpi.com/article/10.3390/ijerph19010430/s1, File S1: SPSS file.
