# Correction: Meh et al. Reliability and Validity of Slovenian Versions of IPAQ-SF, GPAQ, and EHIS-PAQ for Assessing Physical Activity and Sedentarism of Adults. *Int. J. Environ. Res. Public Health* 2022, *19*, 430

**DOI:** 10.3390/ijerph21040417

**Published:** 2024-03-29

**Authors:** Kaja Meh, Vedrana Sember, Saša Đurić, Henri Vähä-Ypyä, Paulo Rocha, Gregor Jurak

**Affiliations:** 1 Faculty of Sports, University of Ljubljana, 1000 Ljubljana, Slovenia; vedrana.sember@fsp.uni-lj.si (V.S.); gregor.jurak@fsp.uni-lj.si (G.J.); 2 Liberal Arts Department, General Education, American University of the Middle East, Egaila 54200, Kuwait; sasa.duric@aum.edu.kw; 3 UKK-Institute, 33500 Tampere, Finland; henri.vaha-ypya@ukkinstituutti.fi; 4 Portuguese Institute of Sport and Youth, 1250-190 Lisbon, Portugal; paulo.rocha@ipdj.pt

Error in Figure/Table

In the original publication [1], there was a mistake in Table 1. Descriptive statistics of the sample; Table 2. Test-retest reliability of IPAQ-SF, GPAQ, and EHIS-PAQ; Table 3. Concurrent validity of IPAQ-SF, GPAQ, and EHIS-PAQ; Table 4. Criterion validity of IPAQ-SF, GPAQ, and EHIS-PAQ against accelerometer UKK RM42; and Figure 1. Bland-Altman plots for the PAQs and UKK RM42 accelerometer according to sedentary behavior and MVPA (min/day) with 95% limit of agreement as published. Due to an error in data coding, the calculation of the time spent in moderate-to-vigorous aerobic recreational activity from EHIS-PAQ was too high. This led to enormous differences in the self-reported PA between physical activity questionnaires. The change in self-reported physical activity also changed the reliability and validity factors for EHIS-PAQ. The corrected Table 1. Descriptive statistics of the sample; Table 2. Test-retest reliability of IPAQ-SF, GPAQ, and EHIS-PAQ; Table 3. Concurrent validity of IPAQ-SF, GPAQ, and EHIS-PAQ; Table 4. Criterion validity of IPAQ-SF, GPAQ, and EHIS-PAQ against accelerometer UKK RM42; and Figure 1. Bland-Altman plots for the PAQs and UKK RM42 accelerometer according to sedentary behavior and MVPA (min/day) with 95% limit of agreement appear below. The authors state that the scientific conclusions are unaffected. This correction was approved by the Academic Editor. The original publication has also been updated.

Text Correction

There was an error in the original publication. The time spent in moderate-to-vigorous aerobic recreational activity from EHIS-PAQ was too high due to an error in data coding. Our data were incorrectly coded as minutes per day, even though participants self-reported their activity in minutes per week when using EHIS-PAQ. This led to enormous differences in the self-reported PA between physical activity questionnaires and to the erroneous conclusion that most over-reporting of physical activity occurred when using EHIS-PAQ. Therefore, we mostly corrected the data in the text.

Several corrections have been made to 3. Results. First correction in paragraph number 3.

Reliability was tested using the test-retest method (Table 2). All results were statistically significant (*p* ≤ 0.001) and showed “low to high” correlations between measurements. With the exception of IPAQ-SF MPA, GPAQ work VPA, and leisure MPA, all correlations were higher than 0.5 and were therefore considered “moderate” [37]. IPAQ-SF and GPAQ had the highest correlations for sedentary behavior (Spearman’s ρ = 0.808 and 0.814, respectively). For EHIS-PAQ, we found the highest test-retest correlations for cycling (Spearman’s ρ = 0.809). For each PAQ, we assessed internal consistency with the Cronbach’s alpha coefficient twice, with and without the sedentary behavior question. The IPAQ showed a “rather reliable” correlation with the sedentary behavior question (Cronbach’s α = 0.297) and a “reliable” without it (Cronbach’s α = 0.685). Similar results were found for the GPAQ: “rather reliable” with the sedentary behavior question (Cronbach’s α = 0.235) and “reliable” without it (Cronbach’s α = 0.669). We found no large differences in EHIS-PAQ, when we excluded the sedentary behavior question. Internal consistency was “rather reliable” with the sedentary behavior question (Cronbach’s α = 0.304) and stayed “rather reliable” without it (Cronbach’s α = 0.310).

Second corrections in paragraph number 4.

The results of the concurrent validity are presented in Table 3. Most of the correlations between the PAQs were very low to low, with some exceptions. For example, sedentary behavior was highly and statistically significantly correlated (Spearman’s ρ = 0.777, 0.772, and 0.857). VPA was moderately correlated (Spearman’s ρ = 0.634), as was MVPA (Spearman’s ρ = 0.597) between IPAQ-SF and GPAQ. There were more relevant and “low to moderate” correlations between GPAQ and IPAQ-SF (e.g., MPA, VPA). In contrast, EHIS-PAQ had no “moderate” correlations with either of the other two PAQs for physical activity, except for the walking item, which correlated with IPAQ-SF walk. “Close to moderate” correlations were found for transport related physical activity (GPAQ transport/EHIS-PAQ walk Spearman’s ρ = 0.497, IPAQ-SF walk/EHIS-PAQ walk = 0.558).

Third corrections in paragraph number 5.

The criterion validity correlations between the PAQs and the UKK RM42 accelerometer are presented in Table 4. We found “low to moderate” and significant correlations for VPA and sedentary behavior. For GPAQ, leisure-time VPA correlated Spearman’s ρ = 0.534 with UKK RM42 while VPA = 0.415. IPAQ-SF VPA had Spearman’s ρ = 0.342 with VPA measured by UKK RM42, while EHIS-PAQ showed no statistically significant correlations for moderato to vigorous recreational activity. For sedentary behavior, IPAQ-SF Spearman’s ρ was 0.454 while for GPAQ was 0.4 and EHIS-PAQ was 0.376.

Fourth corrections in paragraph number 6.

With Bland-Altman plot (Figure 1), we present the differences between data on MVPA and sedentary behavior collected with the UKK RM42 accelerometer and PAQs. One-sample T-tests revealed significant differences between the accelerometers and all three questionnaires for sedentary behavior and MVPA for IPAQ-SF and GPAQ (GPAQ: *p* < 0.000, IPAQ-SF MVPA: *p* < 0.002). Participants underestimated their sedentary behavior on all three PAQs. For sedentary behavior, average difference was lowest for EHIS-PAQ at 125 ± 169 min, while participants underestimated their sitting time by about 2.5 h with IPAQ-SF and GPAQ (IPAQ-SF = 157 ± 160 min; GPAQ = 151 ± 172 min). On the other hand, participants overestimated their MVPA with IPAQ-SF and GPAQ; the average difference for the IPAQ-SF was 17 ± 92 min, followed by GPAQ with 64 ± 143 min). With EHIS-PAQ, participants on average underestimated their moderate to vigorous recreational activity , but the difference was close to zero (−9 ± 64 min on average).

Fifth corrections in paragraph number 7.

According to the WHO guidelines on physical activity and sedentary behavior, 92.5% of participants achieved the recommended amount of MVPA. The figures were lower for the PAQs; 89.5% of participants were considered sufficiently active for IPAQ-SF, 87.3% for GPAQ and 48% for EHIS-PAQ.

Several corrections have been made to 4. Discussion. First correction in paragraph number 1.

This study examined the reliability and validity of the Slovenian versions of EHIS-PAQ, IPAQ-SF, and GPAQ. The main finding of the study is that the most valid and reliable constructs in all tested PAQs were sedentary behavior and VPA, but the criterion validity of these constructs was low (Spearman’s ρ = 0.38–0.45 for sedentary behavior and 0.34–0.42 for VPA). The second important finding is that participants over-reported MVPA for 17 to 64 min and underreported the sedentary behavior for more than two hours with selected PAQs. Third, the GPAQ generally showed the highest criterion validity among observed PAQs, especially for VPA (Spearman’s ρ = 0.415).

Second corrections in paragraph number 5.

Despite slightly higher correlations compared to previous studies using the same instruments, our results showed that participants overestimated their MVPA with IPAQ-SF and GPAQ, but not with EHIS-PAQ (moderate to vigorous recreational activity reported). It has been previously proven that participants tend to over-report physical activity when using PAQs [45]. The systematic review of this problem found an average MVPA overestimation of 106%, when using IPAQ-SF [45], while our result found an overestimation of 29% when using IPAQ-SF, with GPAQ overestimation was higher, but on the other hand participants underestimated their PA when using EHIS-PAQ. WHO recommends 150 min of MVPA per week for adults to achieve significant health benefits [46]. According to our study, 92.5% of participants achieved this amount of MVPA based on the UKK RM42, while 89.5% of participants were sufficiently active according to the IPAQ-SF self-assessment, 87.3% according to the GPAQ, and 48% according to EHIS-PAQ. These overall low discrepancies indicate that PAQs are an appropriate tool for national physical activity surveillance systems to determine the level of physical activity in the population. However, problems with validity and over=reporting should be considered when examining individual physical activity behavior. Several previous studies have already pointed out the problem of over-reporting [47,48] and validity [14,30]; therefore, the results of PAQs should not be used as a measure of individuals’ health-related physical activity without additional information from objective measures.

And a final correction in paragraph number 6.

Our results show greater similarity between IPAQ-SF and the GPAQ, as the concurrent validity between the two is the highest and both have similar criterion validity. Although EHIS-PAQ was developed specifically for the EU and its member states to outdo the disadvantages of PAQs [49], its results in our study on the Slovenian population were not better (MVPA/sport participation = 0.063) and lower compared to the results of the criterion validity study results from Germany, which used ActiGraph GT3X (MVPA = 0.32). Nevertheless, we believe that the question about “moderate to vigorous aerobic recreational activity” and the specific question about muscle-strengthening activities are important for physical activity surveillance. Muscle-strengthening activities are included in the WHO physical activity recommendations [46] and are not included in the most popular and widely used PAQs. The “moderate to vigorous aerobic recreational activity” item highly correlated with leisure physical activity from the GPAQ, once again demonstrating the need for domain-specific questions in PAQs.

The authors state that the scientific conclusions are unaffected. This correction was approved by the Academic Editor. The original publication has also been updated.

## Figures and Tables

**Figure 1:**
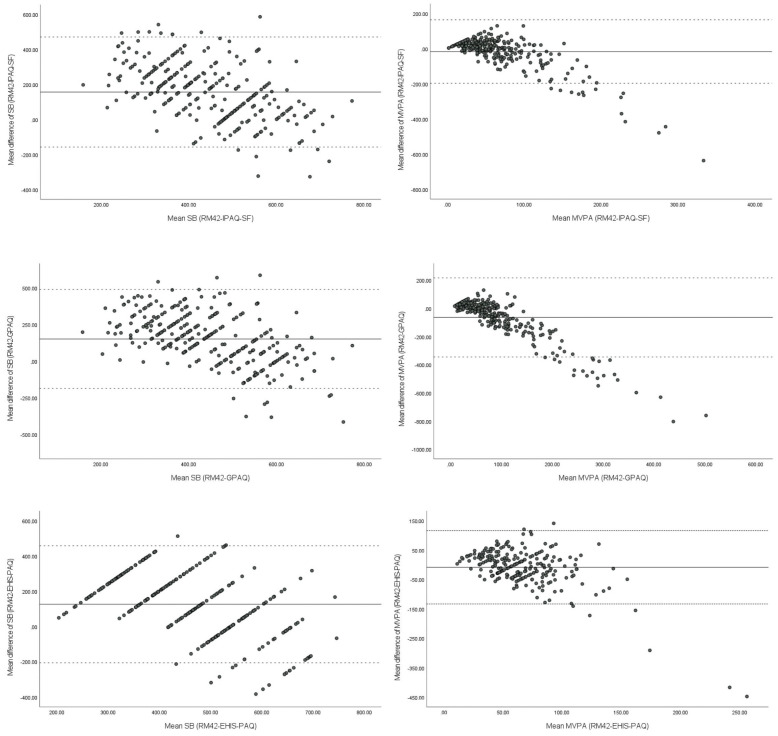
Bland-Altman plots for the PAQs and UKK RM42 accelerometer according to sedentary behavior and MVPA (min/day) with 95% limit of agreement.

**Table 1:** Descriptive statistics of the sample.

		Male		Female		Total	
Age Groups (N)	18–34	38	31.7%	60	32.3%	98	32.0%
	35–49	62	51.7%	87	46.8%	149	48.7%
	50–64	14	11.7%	21	11.3%	35	11.4%
	65–84	6	5.0%	18	9.7%	24	7.8%
BMI Category (N)	<18.5	0	0.0%	3	1.9%	3	1.2%
	18.5–24.9	45	43.7%	101	64.3%	146	56.2%
	25–29.9	42	40.8%	39	24.8%	81	31.2%
	>30	16	15.5%	14	8.9%	30	11.5%
MPA (min/week)	UKK RM42	451.7 (191.4)		364.0 (180.9)		405.3 (190.5)	
	IPAQ-SF	277.2 (397.6)		261.3 (471.4)		266.8 (442.6)	
	GPAQ	542.9 (780.2)		602.0 (828.8)		577.8 (807.9)	
VPA (min/week)	UKK RM42	50.5 (65.7)		34.9 (47.0)		42.2 (56.9)	
	IPAQ-SF	310.2 (389.7)		191.8 (242.4)		237.3 (313.2)	
	GPAQ	407.3 (555.9)		189.9 (282.8)		277.2 (425.6)	
MVPA (min/week)	UKK RM42	455.5 (209.1)		351.2 (178.6)		392.1 (197.5)	
	IPAQ-SF	587.4 (693.3)		453.1 (586.6)		504.9 (632.5)	
	GPAQ	950.1 (181.1)		791.9 (896.3)		854.4 (1019.3)	
	EHIS-PAQ	263.1 (225.8)		230.1 (204.9)		243.5 (213.8)	
Sitting (min/day)	UKK RM42	531.2 (105.5)		513.8 (103.0)		520.6 (104.2)	
	IPAQ-SF	362.0 (188.9)		372.2 (165.6)		366.4 (175.7)	
	GPAQ	369.0 (193.9)		385.9 (172.5)		370.1 (182.4)	
	EHIS-PAQ	405.6 (183.2)		395.6 (172.1)		395.8 (175.3)	

**Table 2:** Test-retest reliability of IPAQ-SF, GPAQ, and EHIS-PAQ.

			Retest																	
			IPAQ-SF						GPAQ								EHIS-PAQ			
			VPA	MPA	Walk	SB			Work VPA	Work MPA	Transport	Leisure VPA	Leisure MPA	SB			Walk	Cycle	MV Aerobic Recreational Activity	SB
Test	IPAQ-SF	VPA	0.648 *				GPAQ	Work VPA	0.466 *						EHIS-PAQ	Walk	0.671 *			
		MPA		0.461 *				Work MPA		0.626 *						Cycle		0.809 *		
		Walk			0.566 *			Transport			0.673 *					SP			0.472 *	
		SB				0.808 *		Leisure VPA				0.764 *				SB				0.694 *
								Leisure MPA					0.424 *							
								SB						0.814 *						

* *p* ≤ 0.001. All results are presented as minutes/day. Notes: IPAQ-SF = International Physical Activity Questionnaire—Short Form; GPAQ = Global Physical Activity Questionnaire; EHIS-PAQ = European Health Interview Survey—Physical Activity Questionnaire; VPA = vigorous physical activity; MPA = moderate physical activity; SP = sport participation; SB = sedentary behavior; MV = moderate to vigorous.

**Table 3:** Concurrent validity of IPAQ-SF, GPAQ, and EHIS-PAQ.

		GPAQ									IPAQ-SF				
		Work VPA	Work MPA	Transport	Leisure VPA	Leisure MPA	MPA	VPA	MVPA	SB	VPA	MPA	Walk	MVPA	SB
EHIS-PAQ	Walk	0.089	0.142 *	0.497 **	0.077	0.264 **	0.219 **	0.133 *	0.227 **	−0.239 **	0.114 *	0.118 *	0.558 **	0.140 *	−0.194 **
	Cycle	0.029	0.063	0.422 **	0.118 *	0.147 *	0.118 *	0.105	0.117 *	−0.145 *	0.092	0.216 **	0.120 *	0.187 **	−0.154 **
	MV aerobic recreational activity	0.122	0.062	0.021	0.175 *	0.001	0.034	0.180 *	0.158 *	−0.001	0.287 **	0.0095	0.025	0.249 **	−0.013
	SB	−0.158 **	−0.321 **	−0.145 *	0.061	−0.208 **	−0.343 **	−0.035	−0.307 **	0.777 **	−0.088	−0.170 **	−0.178 **	−0.189 **	0.772 **
IPAQ-SF	VPA	0.324 **	0.252 **	0.158 **	0.541 **	0.183 **	0.277 **	0.634 **	0.506 **	−0.159 **					
	MPA	0.244 **	0.361 **	0.183 **	0.164 **	0.373 **	0.450 **	0.292 **	0.483 **	−0.235 **					
	Walk	0.137 *	0.262 **	0.382 **	0.024	0.322 **	0.380 **	0.130 *	0.363 **	−0.215 **					
	MVPA	0.341 **	0.392 **	0.187 **	0.376 **	0.318 **	0.446 **	0.523 **	0.597 **	−0.241 **					
	SB	−0.207 **	−0.374 **	−0.169 **	0.042	−0.151 **	−0.356 **	−0.089	−0.329 **	0.857 **					

* *p* ≤ 0.05; ** *p* ≤ 0.01. All results are presented as minutes/day. Notes: IPAQ-SF = International Physical Activity Questionnaire—Short Form; GPAQ = Global Physical Activity Questionnaire; EHIS-PAQ = European Health Interview Survey—Physical Activity Questionnaire; VPA = vigorous physical activity; MPA = moderate physical activity; SB = sedentary behavior; MV = moderate to vigorous.

**Table 4:** Criterion validity of IPAQ-SF, GPAQ, and EHIS-PAQ against accelerometer UKK RM42.

	IPAQ-SF						GPAQ									EHIS-PAQ			
		VPA	MPA	Walk	MVPA	SB	Work VPA	Work MPA	Transport	Leisure VPA	Leisure MPA	MPA	VPA	MVPA	SB	Walk	Cycle	MV Aerobic Recreational Activity	SB
UKK RM42	VPA	0.342 **	0.176 **	−0.043	0.262 **	0.090	0.065	−0.063	0.109	0.534 **	0.119 *	0.010	0.415 **	0.165 **	0.115 *	0.042	0.102	0.067	0.114 *
	MPA	0.258 **	0.179 **	0.313 **	0.243 **	−0.143 *	0.050	0.118 *	0.293 **	0.291 **	0.177 **	0.181 **	0.249 **	0.234 **	−0.115 *	0.261 **	0.125 *	0.071	−0.153 **
	MVPA	0.319 **	0.209 **	0.304 **	0.289 **	−0.132 *	0.060	0.094	0.305 **	0.380 **	0.195 **	0.180 **	0.321 **	0.263 **	−0.098	0.257 **	0.142 *	0.063	−0.129 *
	SB	−0.049	−0.202 **	−0.233 **	−0.169 **	0.454 **	−0.083	−0.324 **	−0.115 *	0.070	−0.225 **	−0.350 **	0.009	−0.280 **	0.400 **	−0.151 **	−0.093	0.08	0.376 **

* *p* ≤ 0.05; ** *p* ≤ 0.01. All results are presented as minutes/day. Notes: RM42 = RM42 triaxial accelerometer; IPAQ-SF = International Physical Activity Questionnaire—Short Form; GPAQ = Global Physical Activity Questionnaire; EHIS-PAQ = European Health Interview Survey—Physical Activity Questionnaire; VPA = vigorous physical activity; MPA = moderate physical activity; MVPA = moderate to vigorous physical activity; SB = sedentary behavior; MV = moderate to vigorous.
